# Anaerobic oxidation of methane coupled with extracellular electron transfer to electrodes

**DOI:** 10.1038/s41598-017-05180-9

**Published:** 2017-07-11

**Authors:** Yaohuan Gao, Jangho Lee, Josh D. Neufeld, Joonhong Park, Bruce E. Rittmann, Hyung-Sool Lee

**Affiliations:** 1 Department of Civil and Environmental Engineering, University of Waterloo, 200 University Ave. W., Waterloo, N2L 3G1 Ontario, Canada; 2 Department of Civil and Environmental Engineering, Yonsei University, Seoul, 120-749 Republic of Korea; 3 Department of Biology, University of Waterloo, 200 University Ave. W., Waterloo, N2L 3G1 Ontario, Canada; 4 Biodesign Swette Center for Environmental Biotechnology, Arizona State University, P.O. Box 875701, Tempe, Arizona 85287-5701 United States of America

## Abstract

Anaerobic oxidation of methane (AOM) is an important process for understanding the global flux of methane and its relation to the global carbon cycle. Although AOM is known to be coupled to reductions of sulfate, nitrite, and nitrate, evidence that AOM is coupled with extracellular electron transfer (EET) to conductive solids is relatively insufficient. Here, we demonstrate EET-dependent AOM in a biofilm anode dominated by *Geobacter* spp. and *Methanobacterium* spp. using carbon-fiber electrodes as the terminal electron sink. The steady-state current density was kept at 11.0 ± 1.3 mA/m^2^ in a microbial electrochemical cell, and isotopic experiments supported AOM-EET to the anode. Fluorescence *in situ* hybridization images and metagenome results suggest that *Methanobacterium* spp. may work synergistically with *Geobacter* spp. to allow AOM, likely by employing intermediate (formate or H_2_)-dependent inter-species electron transport. Since metal oxides are widely present in sedimentary and terrestrial environments, an AOM-EET *niche* would have implications for minimizing the net global emissions of methane.

## Introduction

It is estimated that anaerobic oxidation of methane (AOM) lowers net global emissions of methane (CH_4_) by 10–60%^1^, significantly mitigating the potential impact of this potent greenhouse gas on the global climate. Although certain microorganisms are known to carry out AOM alone, such as *Candidatus* Methylomirabilis oxyfera^2^, AOM also can be more associated with syntrophic microbial interactions. Specifically, anaerobic methanotrophic archaea (ANME) perform AOM in association with sulfate-reducing bacteria (*e.g.*, *Desulfosarcina* and *Desulfococcus*)^3, 4^ or nitrite/nitrate-reducing microorganisms (e.g., *Candidatus* Methylomirabilis oxyfera of the NC10 division and *Kuenenia* anammox population)^2, 5–7^. Thus sulfate, nitrite, or nitrate can serve as electron acceptors for AOM^2, 3, 5, 7^.

The physiological mechanisms underpinning AOM are not fully understood, partly due to the lack of pure cultures. Currently, ANME have been temporarily grouped into three lineages: ANME-1, ANME-2, and ANME-3^8^. These ANME-enriched cultures have certain characteristics in common with methanogens, such as the lipid structures^9, 10^ and the presence of methyl-coenzyme M reductase (MCR)^11^. Available evidence indicates that reverse methanogenesis is one of the main pathways of AOM by ANME^12–15^. In addition to ANME, known methanogens are also able to catalyze AOM^16, 17^, including pure cultures of *Methanobacterium ruminantium*, *Methanobacterium* strain M.o.H., *Methanosarcina barkeri*, and *Methanospirillum hungatii*.

Considering the abundance of iron- and manganese-oxide solids in methane-rich subsurface environments^18, 19^, and the thermodynamic favorableness of metal-dependent AOM reactions, metal-associated AOM may occur naturally in a manner similar to sulfate- and nitrite-dependent AOM and be different from nitrite-AOM or sulfate-AOM. For example, the standard Gibbs free energy at pH 7 (ΔG°′) for AOM using manganese dioxide (MnO_2_) as the terminal electron acceptor is −63.8 kJ/mole e^−^ (Eq. 1), which is about 15-fold higher than that for sulfate-driven AOM (−4.1 kJ/mole e^−^, Eq. 2). ΔG°′ for AOM using ferric hydroxide (Fe(OH)_3_) is −11.1 kJ/mole e^−^, which is still higher than ΔG°′ for sulfate-coupled AOM; in comparison, ΔG°′ for nitrite-AOM is −116 kJ/mole of e^−^.

1 $${{\rm{CH}}}_{4}+4{{\rm{MnO}}}_{2}+7{{\rm{H}}}^{+}\to {{{\rm{HCO}}}_{3}}^{-}+4{{\rm{Mn}}}^{2+}+5{{\rm{H}}}_{2}{\rm{O}}$$

2 $${{\rm{CH}}}_{4}+{{{\rm{SO}}}_{4}}^{2-}\to {{{\rm{HCO}}}_{3}}^{-}+{{\rm{HS}}}^{-}+{{\rm{H}}}_{2}{\rm{O}}$$

Information on metal-oxide-based AOM and on the microorganisms participating in it is minimal compared to sulfate- or nitrite-based AOM^20–24^. Beal and colleagues^20^ identified an increase of sediment *Methanococcoides*/ANME-3 corresponding to MnO_2_-based AOM, and they also found dominant bacteria affiliated with *Bacteroides*, *Proteobacteria*, *Acidobacteria*, and *Verrucomicrobia*. Recent reports demonstrate AOM coupled with metal-oxide reduction^24, 25^, but detailed information is lacking with respect to the pathways or microbial players involved.

Coupling the reduction of metal oxides with AOM implies that AOM is associated with extracellular electron transfer (EET), which is necessary for reducing solid electron acceptors. Recent publications have suggested that EET is potentially involved in AOM between ANME and sulfate-reducing bacteria^26, 27^, where EET is usually unnecessary for utilizing the soluble terminal electron acceptor of sulfate. Although EET is indispensable for using solids as terminal electron acceptors, previous studies^24–27^ have not focused on EET coupled to AOM using metal solids or electrodes as the terminal electron sink. In this study, we provide the direct experimental evidence that AOM may be coupled to EET by using electrodes as the terminal electron sink in a gas-tight microbial electrochemical cell (MxC).

## Materials and Methods

### Microbial Electrochemical Cells (MxCs) and Enrichment of AOM-EET Microorganisms

Dual-chamber MxCs were fabricated with Plexiglas^®^ (Figure S1). The working volumes of the anode and the cathode chambers were 280 mL and 122 mL, respectively. Carbon fibers (24 K carbon tow, Fiber Glast, USA), combined with a stainless steel current collector, were used as the anode; see the literature for detailed procedures for anode construction^28, 29^. Stainless steel mesh (Type 304, McMaster Carr, USA) was used as the cathode. The two chambers were separated by an anion exchange membrane (AEM, AMI-7001S, Membranes International, USA).

Ten mL of return activated sludge (volatile suspended solids of 3.5 g/L) obtained from the Waterloo Wastewater Treatment Plant (August, 2011) was inoculated into a MxC anode chamber. Acetate medium (25 mM) amended with 50 mM phosphate buffered saline (PBS) and other constituents, outlined in Supporting Information (SI), was fed to the anode chamber. The same mineral medium, but lacking acetate, was used for the catholyte (see SI for chemical composition of the medium). The anode potential was fixed at −0.4 V versus an Ag/AgCl reference electrode (−0.2 V against the standard hydrogen electrode (SHE)) (RE-5B, 3 M NaCl, BASi, USA) with a potentiostat (BioLogic VSP, Snowhouse Solutions, Canada). Immediately after inoculation, a nitrogen + carbon dioxide gas (80% N_2_ balanced with CO_2_, Praxair Canada) was used to sparge the anode chamber for 30 min to ensure an anaerobic condition. Subsequently, the MxC was run in fed-batch mode for over four months to grow a biofilm of anode-respiring bacteria.

After the peak current density (7.3 ± 0.4 A/m^2^) was steady in the MxC run in batch mode with acetate (Figure S2), the acetate medium was switched to a methane-saturated medium to stimulate the growth of AOM microorganisms within the biofilm anode. The composition of the methane-saturated medium was the same as the acetate medium, except that acetate was replaced with methane as the sole electron donor and carbon source. We purged the mineral medium in the anode chamber with methane gas (99.97%, Praxair Canada) for two hours, sealed the anode chamber and connected it to a methane gas bag on top-opening. We monitored the dissolved methane concentration with a headspace method^28^ and confirmed saturation of aqueous methane concentration (~ 25 mg CH_4_/L) in the medium during experiments. When the current density decreased to 0.02–0.05 A/m^2^, acetate medium (2 mL) was intermittently injected into the anode chamber (maintaining 0.16–0.42 mmol acetate/L in the anode chamber) to support the growth of EET-dependent bacteria (*e.g.*, *Geobacter*) in the biofilm anode when current density decreased to 0.02–0.05 A/m^2^. As represented by profiles of MxC current density (Figures S2 and S3), we operated the MxC with methane-saturated medium and intermittent acetate spiking for approximately 200 days, then solely with methane medium for over 300 days under continuous methane sparging. To confirm that current generation was from AOM, we ran the MxC by alternating between methane and nitrogen gases (Figure S3).

### Reactor Operation

After ensuring that consistent current generation was from AOM, we placed the MxC inside an anaerobic chamber (Coy Type B Vinyl, Mandel Scientific, Canada) to exclude any possible effects of O_2_ permeation on AOM, considering any O_2_ permeation to the biofilm anode through the Plexiglas or tubing might stimulate aerobic methanotrophs. The H_2_ partial pressure in the anaerobic chamber was constant at 5%. We then operated the MxC using the methane medium in the O_2_-free environment for ~120 days, and the anode potential was fixed at −0.4 V versus the Ag/AgCl reference electrode (−0.2 V vs SHE); the MxC run inside the anaerobic chamber is called the MxC_AC_. To maintain methane-saturation in the anode chamber of the MxC_AC_, we circulated pure methane (99.97%; Praxair) between a glass bottle (500 mL, Kimble, Cole-Parmer) of a gas reservoir and the anode chamber at a flow rate of 7 mL/min using a digital pump (Masterflex L/S Variable-Speed, Cole-Parmer; Figure S4). We refreshed the methane reservoir bottle with pure methane when the current density declined below ~6 mA/m^2^. Electric current and potentials were monitored every 1 min with the potentiostat connected to a personal computer.

To exclude abiotic, non-Faradaic current affecting our results, we operated an abiotic electrochemical cell with a methane -saturated mineral medium for 10 days; the configuration of the abiotic electrochemical cell was identical to the previous MxC_AC_ (Figure S1). We report current density in the MxC_AC_ after subtracting the abiotic current density (1 ± 0.2 mA/m^2^ in Figure S6).

### DNA Extraction, High-throughput Sequencing, and Metagenome Data Analysis

**Figure 1:**
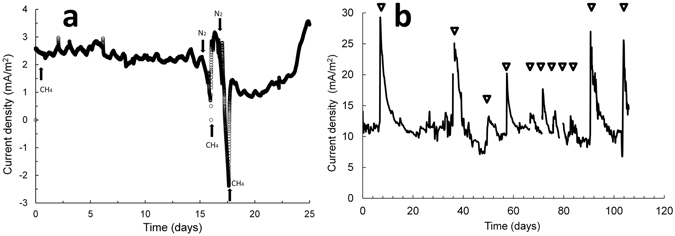
Current generation from anaerobic oxidation of methane in a microbial electrochemical cell (MxC). Methane was the sole electron donor, and no exogenous electron acceptor was provided except for the carbon fiber electrodes. (**a**) Current density in response to alternate methane and nitrogen gas. This alternating gas test was conducted with the MxC in the acclimation period. (**b**) Current density over time in the MxC operated inside the anaerobic chamber (MxC_AC_). Connection and disconnection of the electrodes during the supply of methane caused abrupt increases in current density due to biofilm capacitance effects^42^. Triangles indicate the points of interruption.

We collected a biofilm sample at the end of the experiments (11 cycles of methane gas feed in Fig. 1b, 1,052 days from the beginning of the test) by cutting carbon fibers having biofilms with sterilized scissors in the anaerobic chamber. All of the carbon fibers were cut into small pieces (1–2 cm) and suspended in PBS in 50 ml falcon tubes. The tubes were vortex mixed for 2 min at the highest speed to detach the biofilm, which was then collected as cell pellets in multiple 1.5 ml centrifuge tubes. Genomic DNA (gDNA) was extracted from the biofilm anode from the MxC_AC_ with the PowerSoil DNA Isolation Kit (MO BIO Laboratories, Inc., Carlsbad, USA) according to the manufacturer’s protocol (SI provides the procedure for biofilm collection). Sequencing libraries were prepared by the manufacturer’s protocol of TruSeq DNA PCR-free Sample Preparation Kit (Illumina, Inc., San Diego, CA, USA). For this, 1 µg of genomic DNA was fragmented by adaptive focused acoustic technology (AFA; Covaris). The DNA fragments were end-repaired, size-selected, A-tailed to the 3′ end, and ligated to Illumina adapters^34^. Paired-end sequencing was performed by Macrogen (Seoul, Republic of Korea) using the HiSeq2000 platform (Illumina, San Diego, USA). Approximately 5 Gb of paired-end reads were generated for the sample.

Paired-end reads of 100-base length were uploaded to the MG-RAST server (IDs: 4616918.3)^35^. The paired-end read datasets were merged and filtered using default pipeline options, which included removal of reads that were artificial replicates, affiliated with *Homo sapiens* DNA (NCBI v36), associated with a Phred score less than 15, or reads with more than five low-quality bases. Archaeal and bacterial taxa were assigned using Best Hit Classification at an E-value cutoff of 10^−5^, minimum identity cutoff of 97%, and a minimum alignment length of 50 bases using the SILVA Small Subunit (SSU) rRNA database^30, 31^. Functions of AOM were annotated by Hierarchical Classification based on KEGG Orthology database at an E-value cutoff of 10^−5^, a minimum identity threshold of 60%, and a minimum alignment length of 17 amino acids^32^. To characterize taxa for each enzyme in an AOM pathway, AOM-related nucleotide sequences were selected and downloaded from the Hierarchical Classification results. Protein sequences for construction of AOM databases were extracted from the Genbank. The BLASTx analysis was performed with standalone BLAST software with the selected nucleotide sequences and the constructed databases of AOM. The EET-related enzymes were annotated using “All Annotations” tool, based on KEGG annotations with an E-value cutoff of 10^−5^, minimum identity cutoff of 60%, and a minimum alignment length of 17 amino acids^32^.

### Fluorescence *in situ* Hybridization

At the end of the experiments (1,202 days from the beginning of the test), we cut carbon fibers with sterilized scissors in the anaerobic chamber for FISH assays. We used 16S rRNA-targeted oligonucleotide probes (Table S1) for visualizing biofilms comprising the *Methanobacteriaceae* family and *Geobacter* genera in the MxC_AC_. The probe for the *Methanobacteriaceae* family was labeled with fluorescein (green fluorescence) and the probes for the *Geobacter* genus was labeled with TAMRA (red fluorescence). Carbon fibers were fixed with 2% paraformaldehyde and 0.5% glutaraldehyde in 50 mM PIPES for one hour, washed with PBS, and then fixed on gelatin-coated glass slides. Details of the FISH procedures were previously published^33–35^. We observed hybridized samples with a Zeiss Axiovert 200 microscope equipped with an LSM510-Meta confocal module and a Zeiss Axio Scope.A1 epifluorescence microscope equipped with an AxioCam CM1 camera. The confocal images were extracted using the Carl Zeiss Zen 2012 SP1 (black edition) and the epifluorescence images were extracted using the Carl Zeiss Zen 2012 (blue edition). All images were compiled in the Microsoft Office Visio 2013.

### Carbon Isotopic Analysis

To provide more evidence of an AOM reaction occurring in the MxC_AC_, we analyzed the isotopic composition of carbon dioxide (806 days from the beginning of the test) from the headspace of the MxC_AC_ that was operated with the gas re-circulation loop equipped with a Supelco gas sampling bulb (250 mL, PTFE stopcock; Sigma-Aldrich) (see Figure S4). The top part of a combination valve from a Tedlar sampling bag (Cole-Parmer) was fitted to the sampling port. The original septum in the combination valve was replaced by a GC septum (Restek Thermolite, Mandel Scientific). All connections were sealed with a silicone sealant (Dow Corning 736). The loop and reactor were flushed and filled/saturated with ultra-high-purity methane gas (99.97%) before the incubation, which lasted for 8.9 days until the carbon dioxide inside the loop exceeded 2 mL, which is the recommended minimal volume for downstream gas extraction^36^. A digital pump (Masterflex, L/S, Cole-Parmer) was used to maintain the gas re-circulation rate at 7 mL/min. The gas composition was monitored with a gas chromatography method^37^. The extraction of carbon dioxide and isotopic composition analysis were carried out in the University of Waterloo-Environmental Isotope Laboratory according to previously published protocols^36, 38^. The isotopic composition of the methane gas (99.97%, Praxair) used for the operation of the MxC_AC_ was also analyzed. The fractionation factor of carbon and the ratio of ^13^C to ^12^C were computed, according to the equations provided elsewhere^39^, and the standard carbon isotope atomic ratios ${(\frac{{}^{13}C}{{}^{12}C})}_{\text{standard},\text{PDB}}\,$and ${(\frac{{}^{13}C}{{}^{12}C})}_{\text{standard},\text{VPDB}}\,\,$are 0.011237 and 0.011180 (PDB/VPDB: Pee Dee Belemnite/Vienna Pee Dee Belemnite)^39^, respectively. The stable carbon-isotope signature (δ^13^C) for headspace CO_2_ in the MxC_AC_ and the pure methane used for MxC_AC_ was computed with Eq. 3.

3 $${\delta }^{13}{\rm{C}}=({{\rm{R}}}_{{\rm{Sample}}}/{R}_{{\rm{Standard}}}-1)\times 1000\textperthousand $$

where R is the ratio of ^13^C to ^12^C and R_standard_ is either the PDB standard or the VPDB standard.

## Results and Discussion

### Current Density from AOM in a Biofilm Anode

Our results demonstrate that AOM was coupled with EET to the anode, generating electric current (Fig. 1). The current density in the MxC approached zero or became negative when the MxC was flushed with N_2_ gas (99.999%). However, the current density increased again with the provision of methane gas (Fig. 1a); we confirmed no net current generation from ammonium nitrogen in the MxC_AC_ (see Figure S5). Biofilm anodes can act as a “bio-capacitor,” which means that electron carriers in the biofilm can store electrons or act as a capacitor. The observed negative current during N_2_ sparging suggests that electrons could transfer from the anode to the electron carriers in the biofilms^40–42^. The highest current density was 7.3 ± 0.4 A/m^2^ in the MxC fed with acetate medium. In comparison, the net electric current from AOM ranged from 6.6 to 13.6 mA/m^2^ (average, 11.0 ± 1.3 mA/m^2^) in the MxC_AC_ (Fig. 1b) when non-Faradaic current is taken into account for the abiotic electrochemical cell. Because H_2_ is generated at the cathode, it is possible that a very small amount of H_2_ gas diffused to the anode and might have affected current density in the biofilm anode. Further study would be required to quantify the effect of H_2_ diffusion on AOM-EET in the biofilm anodes. The effect of H_2_ diffusion has to have been very small because the H_2_ generated at the cathode came from the oxidation of methane at the anode. As H_2_ diffusion from the cathode could not have been a significant fraction of the H_2_ generated at the cathode, the relative impact of H_2_ oxidation at the anode would have been negligible.

### Carbon Isotopic Analysis for Headspace Gas in the MxC_AC_

The δ^13^C values of CO_2_ sampled from the headspace of the MxC_AC_ (Table S2) were −56.4‰ and −58.4‰ for duplicate measurements after 8.9 days of incubation. The δ^13^C of pure methane provided to the MxC_AC_ for the corresponding runs were −37.14‰ and −37.23‰. The fractionation factors $({(\frac{{}^{13}C}{{}^{12}C})}_{methane}/{(\frac{{}^{13}C}{{}^{12}C})}_{carbondioxide})$ in the headspace gas were 1.0153 and 1.0174, which are within the range commonly observed for AOM (1.012 to 1.039)^43^. In general, the fractionation factor for methane oxidation reactions is larger than 1; for instance, the factors range from 1.0054 to 1.025 for methane oxidation reactions in the atmosphere, where methane is oxidized by hydroxyl radicals^44^, and are 1.003 to 1.049 in aerobic methane oxidation systems^45, 46^. In comparison, the fractionation factors are relatively smaller in methane-producing environment: 0.924 to 0.979 for hydrogenotrophic methanogenesis^47^. A second isotopic analysis of methane was carried out two months after we supplied pure methane-^13^C for a DNA labeling test; however, the slow metabolism of methane supplied during a DNA-labeling test (nine days, 99% ^13^CH_4_, data not shown) affected the subsequent analysis of the oxidation of regular methane. The δ^13^C of carbon dioxide, +4.110, was much higher than the background values, indicating the consumption of methane. More study on isotopic experiments and DNA-based stable isotope probing experiments over a prolonged period is required to confirm AOM-EET in the biofilm anode. The carbon-isotope results support that methane was oxidized anaerobically in the MxC_AC_, which was operated in the anaerobic chamber.

### Metagenome on the Biofilm Anode

**Figure 2:**
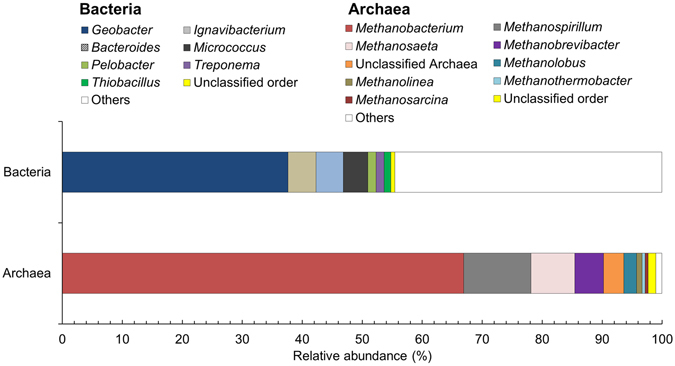
Community composition of bacteria (top) and archaea (bottom) using Best Hit Classification based on the SILVA Small Subunit (SSU) rRNA database in MG-RAST. The total abundance of SSU rRNA gene reads was 27,084: 22,946 reads were bacterial, and 982 reads were archaeal. The category “Others” indicates these genera of archaea and bacteria had relative abundance below 1%.

A metagenome from the MxC_AC_ anode biofilm was represented by ~50 million paired-end reads that were merged, with ~32 million reads passing quality control for annotation (Table S3). Of these, 982 sequences were classified as archaeal small subunit (SSU) rRNA genes, whereas 22,946 sequences were affiliated with bacterial SSU rRNA genes. The majority of archaeal SSU rRNA gene sequences (Fig. 2) corresponded to *Methanobacterium* (67%), with lower contributions of sequences affiliated with *Methanospirillum* (11%), *Methanosaeta* (7%), and *Methanobrevibacter* (5%). The majority of the bacterial SSU rRNA gene sequences corresponded to *Geobacter* (38%), followed by *Ignavibacterium* (5%), *Bacteroides* (4%), and *Micrococcus* (4%). Gene sequences constituting less than 1% of the total sequences are defined as “others.”

**Figure 3:**
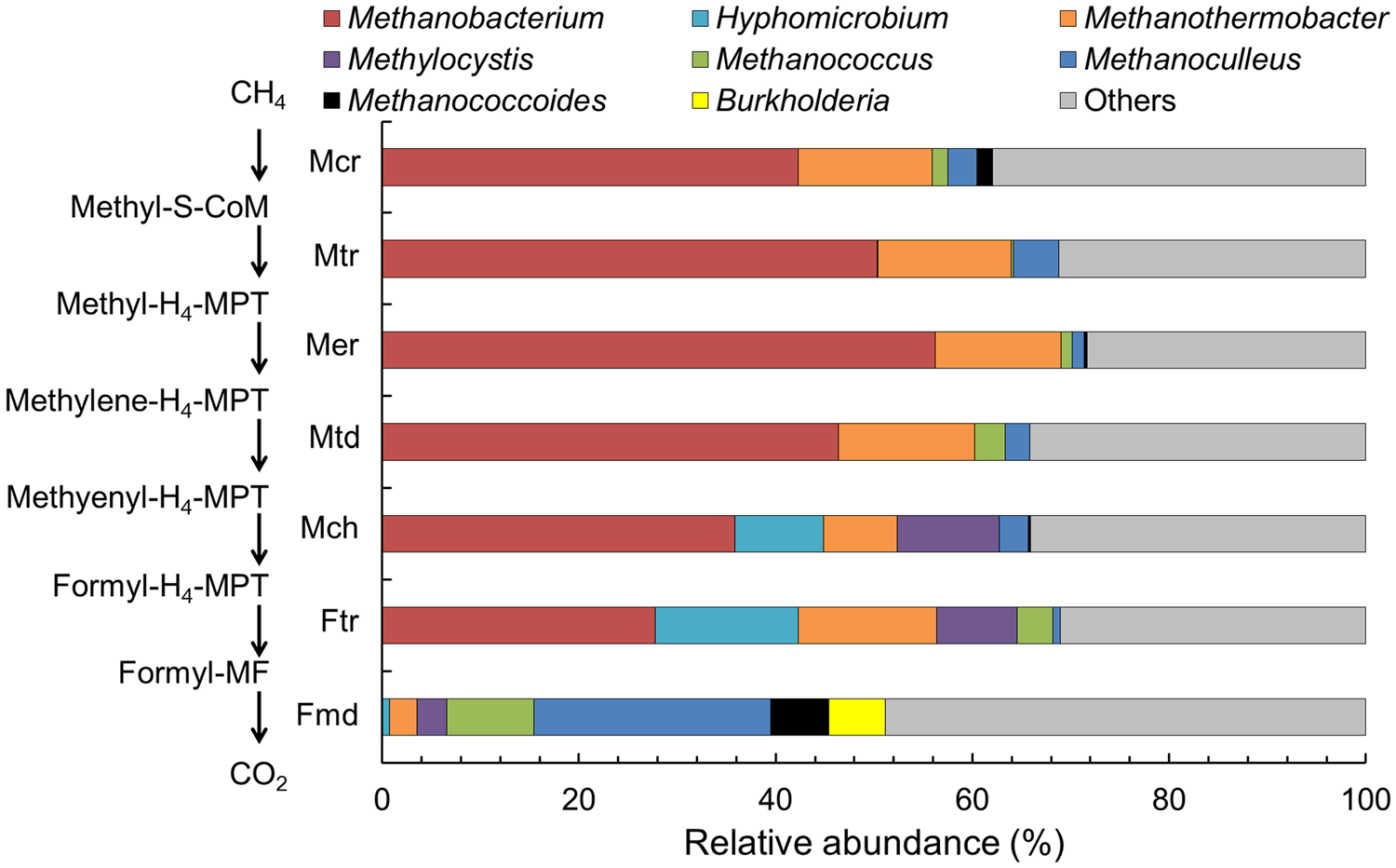
Relative abundances of genes for AOM-related enzymes according to taxonomic annotations by BLAST. The category “Others” indicates genera with relative abundance below 1%.

From functional annotation of the metagenome by hierarchical classification, 63,234 reads were affiliated with methane metabolism. The *pmo* gene, encoding particulate methane monooxygenase, which is mainly essential for aerobic oxidation of methane, was not detected, suggesting that O_2_-dependent methane oxidation would not occur in the MxC, although aerobic methanotrophs (*Methylococcaceae* and *Methylocystaceae*) comprised 0.8% of the classified ribosomal RNA genes (data not shown). In comparison, most of the genes essential for methanogenesis, which also have been identified in AOM, were detected and represented 24.1% of reads annotated to methane metabolism^13^. According to taxonomic identification of the essential AOM-pathway genes detected by shotgun sequencing, the AOM-pathway genes belong to the family *Methanobacteriaceae*, such as *Methanobacterium* and *Methanothermobacter*, but none of these genes were associated with the *Geobacter* genus. Considering the results of SSU rRNA gene data, most AOM genes were affiliated with *Methanobacterium*-related archaea. Previous studies have reported that taxa from this genus can oxidize methane anaerobically^16, 48^, despite the fact that these archaea are distinct from previously known ANME clades^49^ (Fig. 3). Nonetheless, Zehnder and Brock reported that pure cultures of *Methanobacterium ruminantium*, *Methanobacterium* strain M.o.H., *Methanobacterium formicicum, Methanobacterium thermoautotrophicum, Methanobacterium arbophilicum*, and *Methanobacterium* strain AZ oxidized methane anaerobically, probably via reverse methanogenesis^16^, while most of the recent publications have identified ANME for AOM in natural environments^3–8^. Sivan *et al*.^21^ also reported that *Methanosarcina barkeri* switched catabolism from methanogenesis to iron respiration using iron oxide as the terminal electron acceptor and methane as the electron donor, further supporting the capacity of some methanogens to conduct reverse methanogenesis.

**Figure 4:**
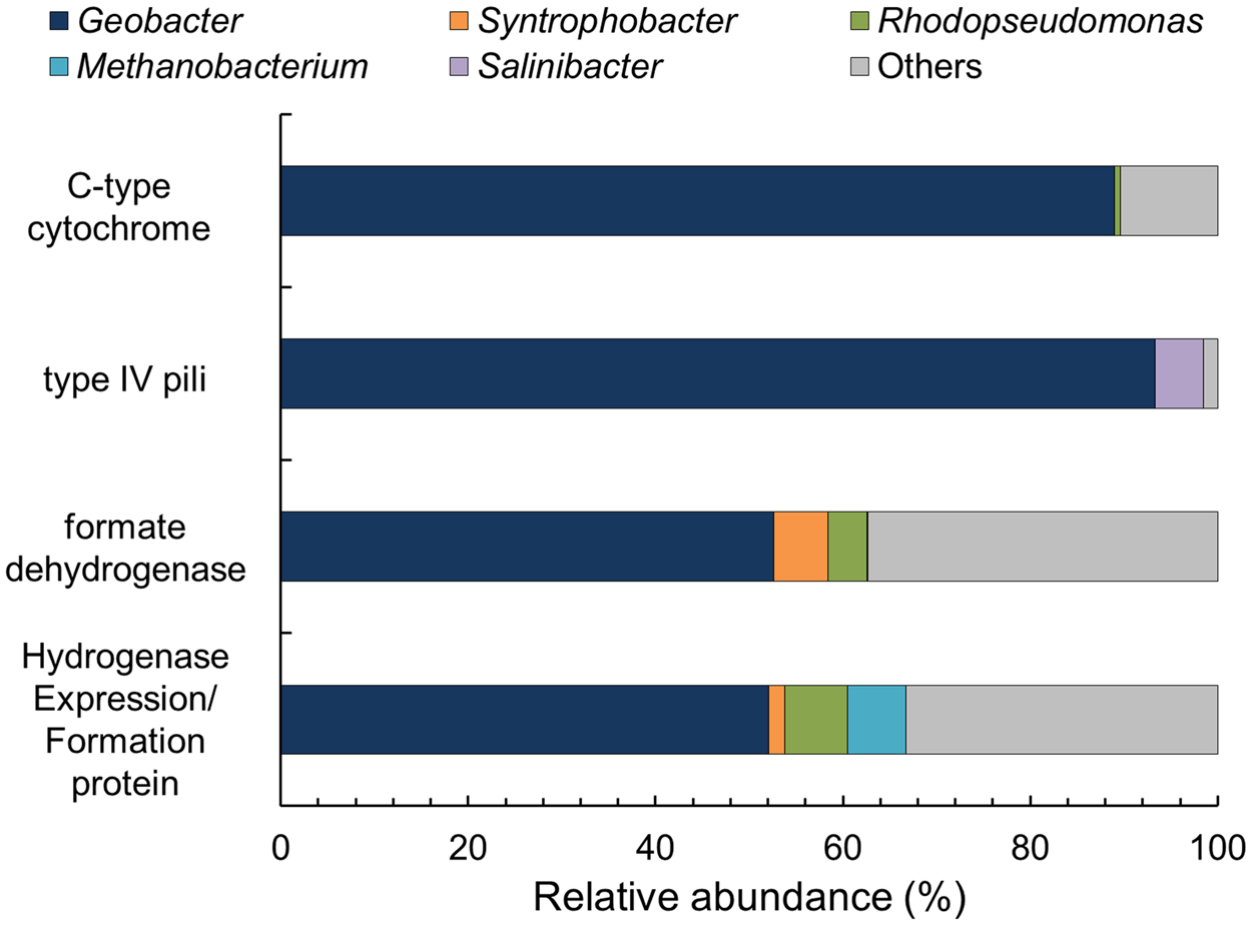
Relative abundances of genes for EET-related enzymes according to taxonomic annotations. The category “Others” indicates genera with relative abundance below 1%.

Genes encoding cytochrome c and type IV pili, which are associated with EET, were found in the metagenome at 0.96% and 0.16% of total functional abundance, respectively (Table S4). *Geobacter*-related bacteria accounted for 89% of cytochrome c annotations and 94% of type IV pili annotations (Fig. 4). In contrast, *Methanobacterium* spp. did not contribute any of these genes to the metagenome. Because members of the *Geobacter* genus have been widely reported as one of the major EET bacteria^50, 51^, these observations imply that the *Methanobacteriaceae* family, especially *Methanobacterium* spp., partnered syntrophically with *Geobacter* spp. to conduct an EET-coupled AOM, not by directly transferring methane-derived electrons to the anode. In this syntrophy, *Methanobacterium* spp. might transfer electrons originating in methane through a form of direct interspecies electron transfer (DIET) via conductive pili-like appendages which contain multi-heme cytochromes, as suggested for *Methanosaeta* and *Geobacter* species or ANME and sulfate-reducing bacteria^26, 27, 52, 53^; however, no direct evidence is available to prove DIET via appendages.

Although the DIET pathway is not confirmed for consortia involving methanogens, literature using metagenomic or metatranscriptomic data indirectly suggests that DIET is possible for these consortia^26, 27, 52, 53^. For instance, the genomes of ANME-2 encoded multi-heme cytochromes important for EET^7, 54^, potentially allowing DIET with sulfate-reducing bacteria. In comparison, no study has reported multi-heme cytochromes in *Methanobacterium* spp., and the metagenome analysis in our study did not detect multi-heme cytochromes for the methanogen, implying that DIET would not occur between *Geobacter* and *Methanobacterium* genera, important players in the biofilm anode. Instead, the metagenome in our study was affiliated with formate dehydrogenase and hydrogenase genes, and over 50% of these genes were annotated as being associated with *Geobacter*-related bacteria. The reads of formate dehydrogenase and hydrogenase genes imply that *Geobacter* spp. might oxidize formate or hydrogen generated from AOM catalyzed by *Methanobacterium* (*i.e.*, reverse methanogenesis). This suggests that the consortium of *Methanobacterium* and *Geobacter* might employ intermediate-dependent interspecies electron transfer to the anode, not DIET.

### Fluorescence *in situ* hybridization (FISH) Images

**Figure 5:**
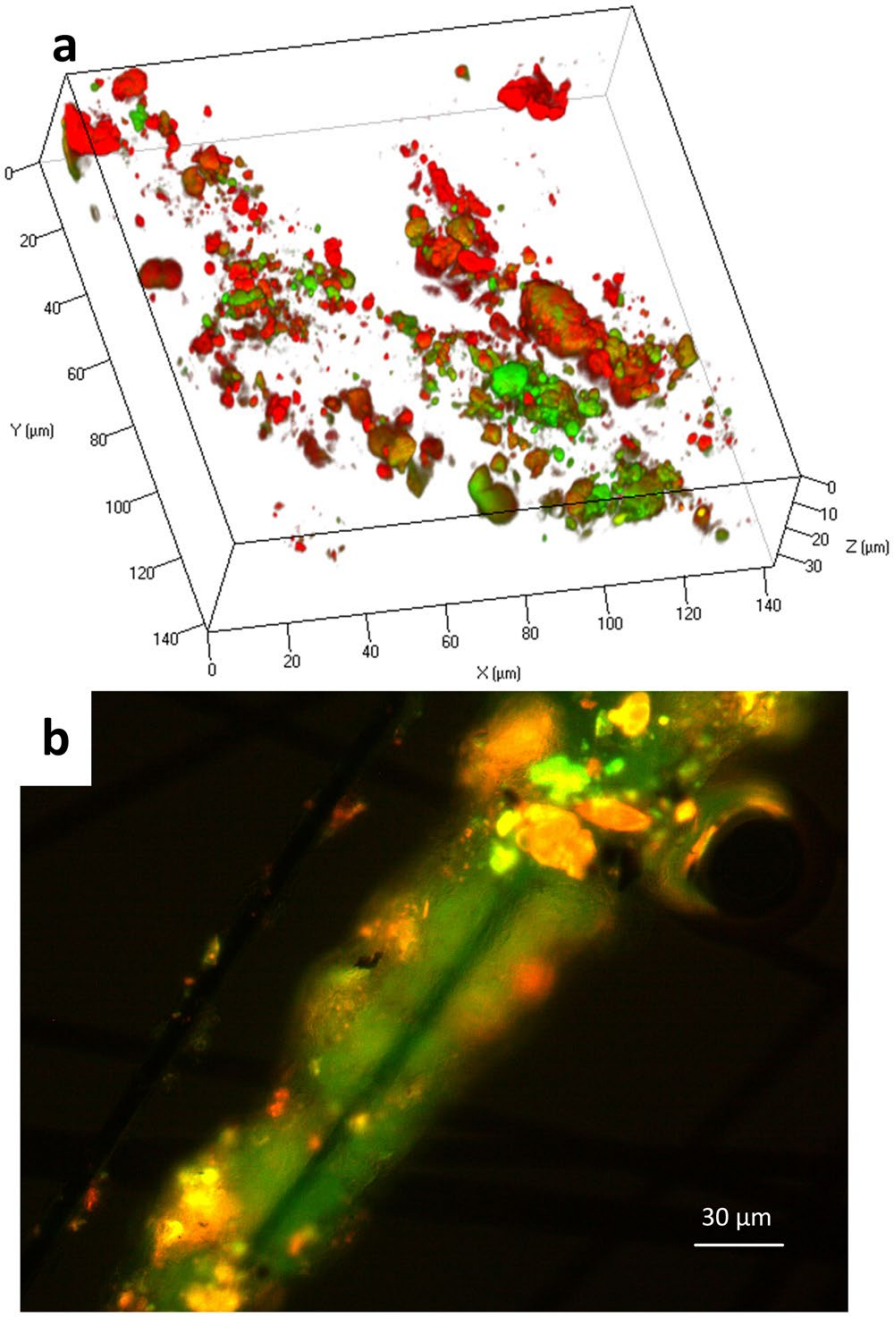
Fluorescence *in situ* hybridization (FISH) images of intact biofilms on carbon fibers from the microbial electrochemical cell anaerobic chamber (MxC_AC_) anode. (**a**) shows the 3D structure of the colonies formed by *Methanobacteriaceae* family (green) and *Geobacter* genus (red) on the surfaces of a single carbon fiber; yellow indicates the overlap of green and red colors. (**b**) shows biofilm structure in 2D. Image a is obtained from a confocal microscope, and image b is from an epifluorescence microscope.

Microscopic analysis, shown in Fig. 5, demonstrated that members of the *Methanobacteriaceae* family (green) and *Geobacter* genus (red) co-existed on the surface of the anode’s carbon fibers, similar to mixed aggregates of ANME-1 and sulfate-reducing *Desulfosarcina* ^55^. These images support the hypothesized syntrophy between *Methanobacterium* spp. and *Geobacter* spp. for enabling the EET-coupled AOM reaction using the anode as a terminal electron acceptor, not direct electron transfer by *Methanobacterium* alone.

### Implication of the AOM Coupled with EET to Electrodes

Several studies reported that methanogens could anaerobically consume methane^16, 17, 48^. However, relatively few study has documented that AOM can be coupled to EET via syntrophy between bacteria and archaea. Our study clearly demonstrates EET-coupled AOM in a biofilm anode, probably via intermediate-dependent (i.e., formate or H_2_) inter-species electron transfer between *Methanobacterium* and *Geobacter*. Future research is needed to understand AOM-EET mechanisms, including a possible DIET mechanism between the two genera.

The evidence from this study implies a new syntrophic interaction between methanogens and bacteria for transferring electrons to metal-oxide solids. On the one hand, this syntrophy is ironic, because methanogens conventionally are viewed as competitors to the bacteria that reduce metal oxides, since they compete for the electron donors (*e.g.*, H_2_)^56, 57^. On the other hand, the new syntrophy could have substantial implications for lowering estimates of global methane flux, because methanogens and metal-oxide solids are widely distributed in sedimentary and terrestrial environments.

## Electronic supplementary material

Supplementary Information
