# Supplementary material for: Anaerobic oxidation of methane coupled with extracellular electron transfer to electrodes
Source: Sci Rep. 2017 Jul 11;7:5099. doi: 10.1038/s41598-017-05180-9 (PMC5506047; doi:10.1038/s41598-017-05180-9)
Supplement: Supplementary file 1 — Supplementary Information [file 41598_2017_5180_MOESM1_ESM.doc]

**Supplementary Information**

**Anaerobic oxidation of methane coupled with extracellular electron transfer to electrodes**

Yaohuan Gao^1^, Jangho Lee^1,2^, Josh D. Neufeld^3^, Joonhong Park^2^, Bruce E. Rittmann^4^, and Hyung-Sool Lee^1^*

^1^ Department of Civil and Environmental Engineering, University of Waterloo, 200 University Ave. W., Waterloo N2L 3G1, Ontario, Canada

^2^ Department of Civil and Environmental Engineering, Yonsei University, Seoul 120-749, Republic of Korea

^3^ Department of Biology, University of Waterloo, 200 University Ave. W., Waterloo N2L 3G1, Ontario, Canada

^4^ Swette Center for Environmental Biotechnology, The Biodesign Institute at ArizonaState University, P.B. Box 875701, Tempe, Arizona 85287-5701, United States of America

*Corresponding author: Phone: +1-519-888-4567 Ext. 31095; Fax: +1-519-888-4349; E-mail: hyungsool@uwaterloo.ca

Email addresses: y55gao@uwaterloo.ca (Yaohuan Gao); jangho.lee@uwaterloo.ca (Jangho Lee); jneufeld@uwaterloo.ca (Josh D. Neufeld); parkj@yonsei.ac.kr (Joonhong Park); Rittmann@asu.edu (Bruce E. Rittmann)

Number of Pages: 12

Number of Figures: 6

Number of Tables: 4

**Composition of Acetate Medium**

The composition of acetate medium consists of 50 mM phosphate buffer (Na_2_HPO_4_/KH_2_PO_4_), 0.7 mM NH_4_Cl, and 25 mM CH_3_COONa, and a mineral solution having the final concentration (in 1L): 5 mg EDTA, 11.6 mg MgCl_2_, 5.9 mg Mn_2_Cl_2_-4H_2_O, 0.8 mg CoCl_2_-6H_2_O, 1.14 mg CaCl_2_-2H_2_O, 0.5 mg ZnCl_2_, 0.1 mg CuSO_4_-5H_2_O, 0.1 mg AlK(SO_4_)_2_, 0.1 mg H_3_BO_3_, 0.2 mg Na_2_MoO_4_-2H_2_O, 0.01 mg Na_2_SeO_3_, 0.1 mg Na_2_WO_4_-2H_2_O, and NiCl_2_-6H_2_O. We autoclaved the medium, let it cool, purged it with N_2_ (99.999%) for 30 min, and added FeCl_2_-2H_2_O (20 μM) and Na_2_S-9H_2_O (77 μM) through a sterile syringe filter (Advantec 0.2 μm, PTFE, Cole-Parmer).

**Biofilm Collection**

Carbon fibers of the MxC were cut with sterilized scissors, and the fibers were then suspended in sterilized phosphate-buffered saline in a 50 mL sterilized plastic Falcon tube. The tube was shaken with a vortex mixer for 2 min at the highest speed to detach the biofilm. The cell suspension was distributed into multiple pre-sterilized 1.5 mL microcentrifuge tubes and centrifuged at 10,000 g for 3 min using a microcentrifuge (Eppendorf 5424, Canada) to collect cell pellets. We repeated this procedure twice to improve biofilm collection and the cell pellets were stored at -80°C prior to further processing.


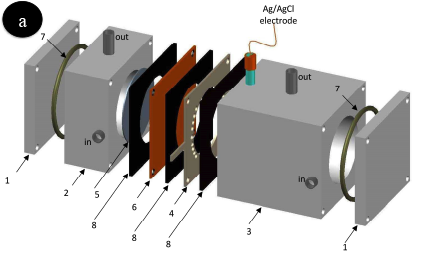


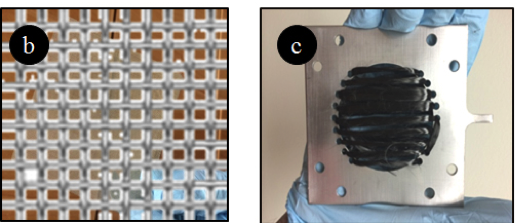


**Figure S1.** The schematic diagram of a gas-tight dual-chamber microbial electrochemical cell (MxC) and the associated electrodes. (a): the MxC, (b): stainless steel mesh cathode, and (c): the anode current collector combined with carbon fibers (courtesy of Dhar et al., 2016). The components of the MxC are 1-cover, 2-cathode chamber, 3-anode chamber, 4-anode current collector (carbon fibers are not shown here), 5-stainless steel cathode mesh, 6-anion exchange membrane, 7-O-ring, and 8-rubber gasket. All parts are pressed together by nuts and screw rods.


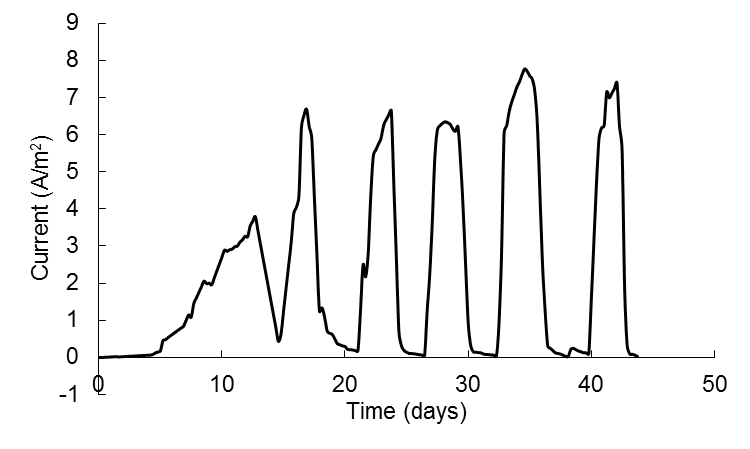


**Figure S2.** The profile of current density in the microbial electrochemical cell (MxC) during initial acclimation phase using 25 mM acetate medium. The MxC was operated in a fed-batch mode.


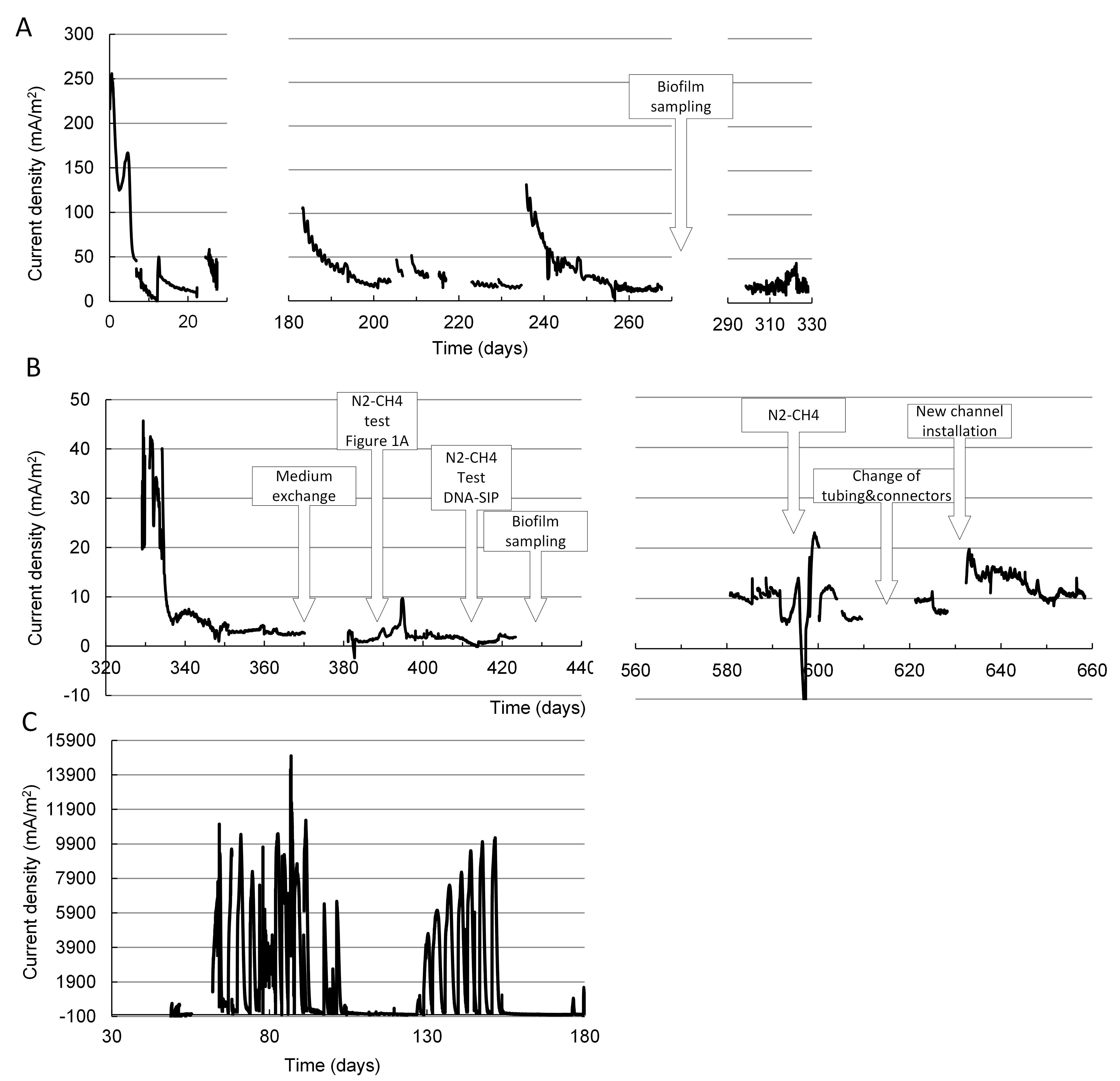


Figure S3. The electric current profile from the microbial electrochemical cell (MxC). A: the current during the acclimation phase; B: the current when only methane was supplied as the sole carbon and energy source; C: the current during day 30 to day 180 when acetate medium was intermittently spiked into the anode chamber. Arrows explain why current gaps were created during the long-term experiments, which include biofilm sampling and biofilm regrowth, medium replacement (bottle, tubing, connectors, etc.), N_2_-CH_4_ alternation tests, DNA-stable isotope probing (SIP) experiments followed by biofilm sampling, and change of a potentiostat channel.

| A 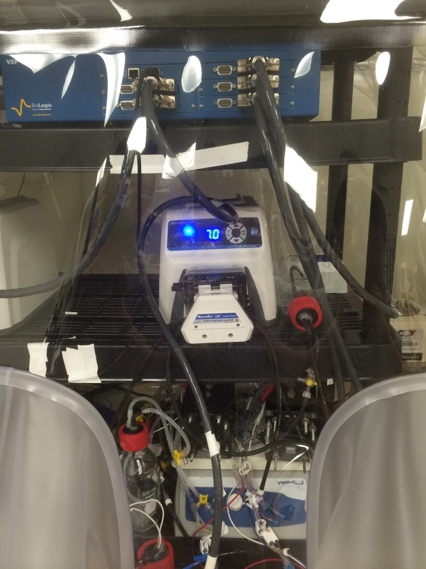 C 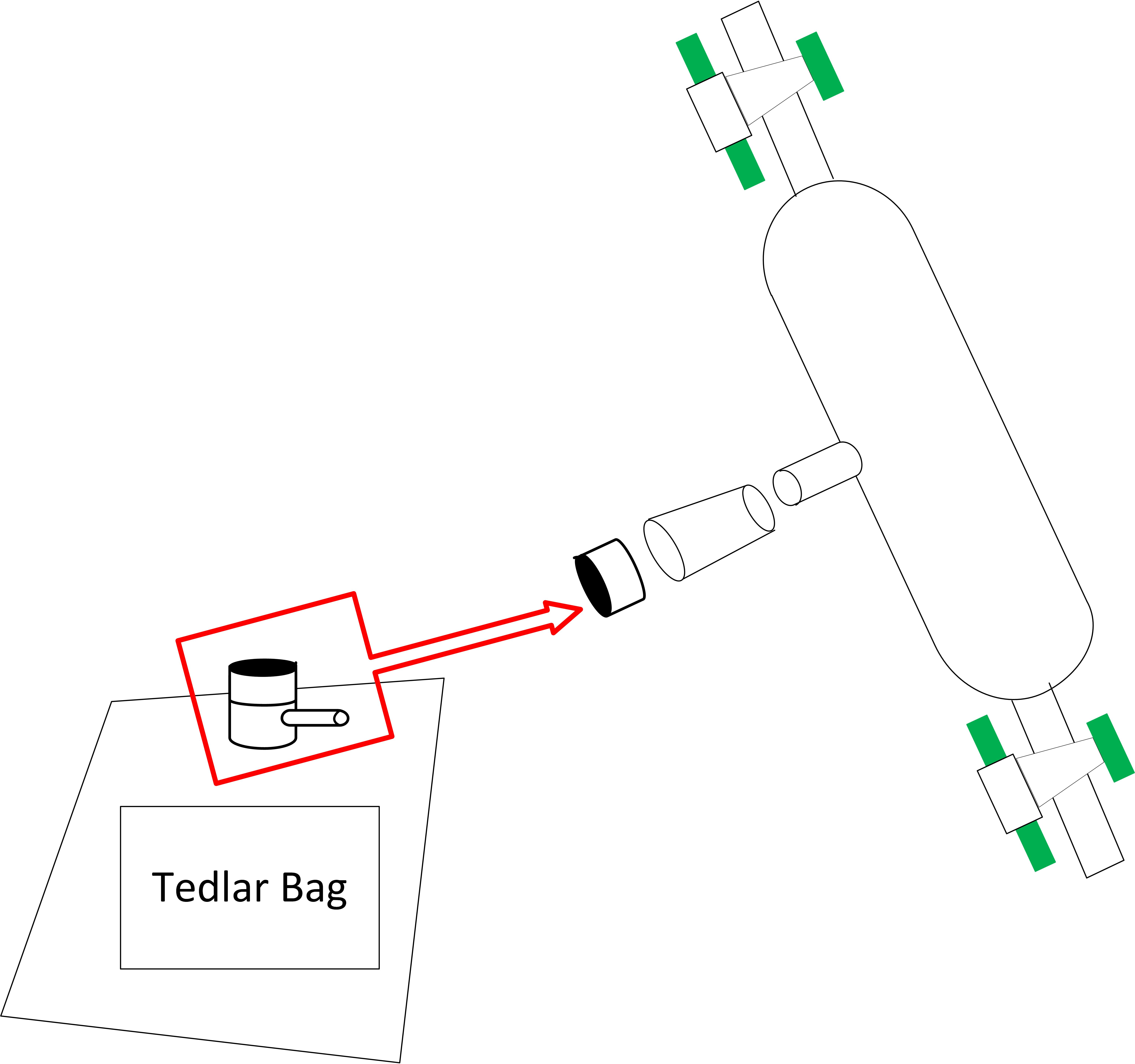 |
| --- |
| B |

**Figure S4.** The schematic of a gas-recirculation loop system for the microbial electrochemical cell operated inside an anaerobic chamber (MxC_AC_). A. the photo of the MxC_AC_ equipped with a loop system inside an anaerobic chamber; B. a schematic of the loop system; and C. a gas sampling bulb used to replace the glass bottle for collecting the headspace gas for carbon isotope analysis. The top part of the combination valve from a Tedlar gas sampling bag was used as the gas sampling port.


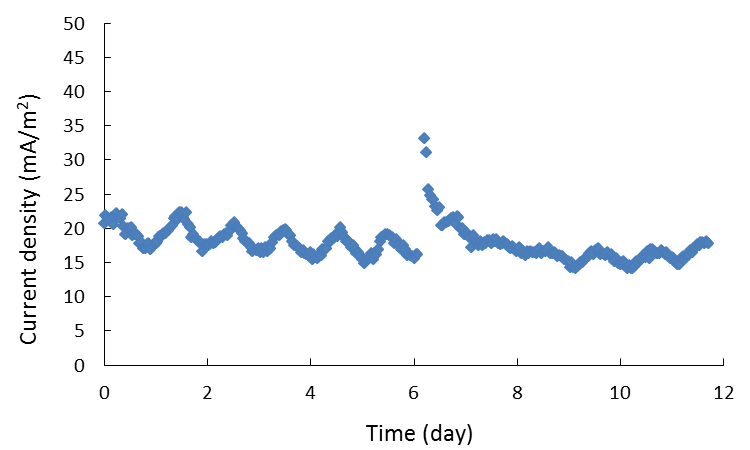


**Figure S5.** The electric current profiles before and after 1 ml of ammonium chloride (NH_4_Cl) solution was injected at day 6. The final concentration of NH_4_Cl in the anolyte was 2.7 mM as NH_4_^+^-N. The sharp increase of current density in day 6 occurred due to the disturbance from the injection of degassed NH_4_Cl solution. The current spike corresponds to less than 2.6% of the available electrons from NH_4_^+^-N. The fluctuation of the electric current was caused by the variation of the temperature in our lab.


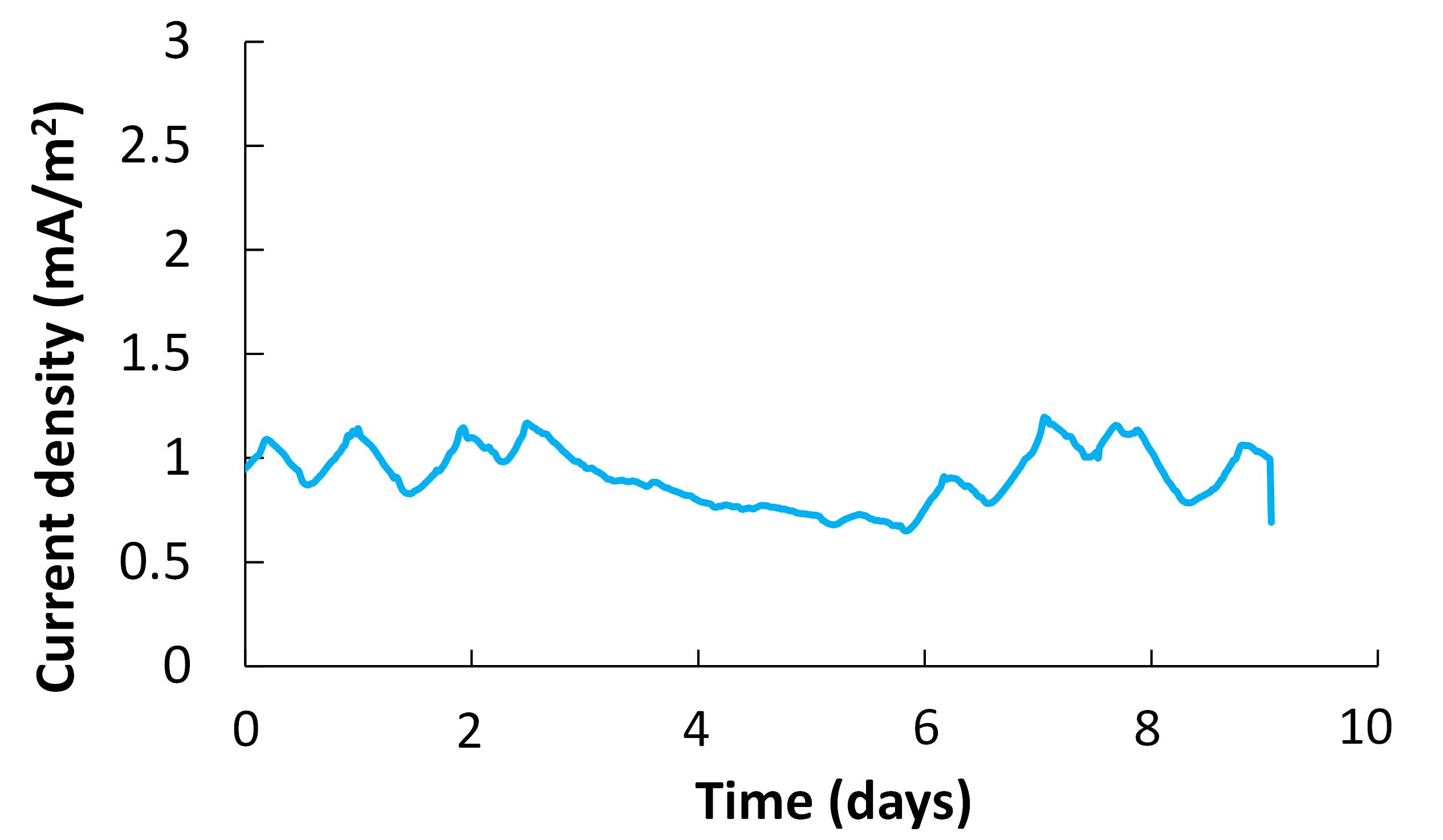


**Figure S6.** The non-Faraday current from an abiotic electrochemical cell at fixed anode potential of -0.4 V vs Ag/AgCl. The electrochemical cell was sterilized with ethanol solution (75 %) and rinsed with DI water. The original but filtrated (0.45 µm syringe filter, Nylon, Cole-Parmer Canada) anolyte lacking methane was used as the anolyte in this test.

Table S1. Fluorescently labeled oligonucleotide probes used in this study

| **Probe name** | **Generic name** | **Target** | **Sequences (5’ to 3’)** | **Reference** |
| --- | --- | --- | --- | --- |
| MB1174 | S-F-Mbac-1174-a-A-22 | *Methanobacteriaceae* | TACCGTCGTCCACTCCTTCCTC | (Rotaru, A.E. et al. 2012) |
| Geo3 a,b, and c | S-G-Geob-0818-a-A-21 | *Geobacter* cluster* | CCGCAACACCTAGTACTCATC | (Richter, H. et al. 2007) |
|  | S-G-Geob-0818-b-A-21 |  | CCGCAACACCTAGTTCTCATC |  |
|  | S-G-Geob-0818-c-A-21 |  | CCGCAACACCTGGTTCTCATC |  |

*the three probes were evenly mixed to give the maximum coverage of the cluster

**Table S2**. Isotope analyses of the carbon dioxide (product) generated from the anode chamber and the methane gas (feed)

| **Sample** | **δ^13^C** | **Result** | **Repeat** | **δ^13^C** | **Result** | **Repeat** |
| --- | --- | --- | --- | --- | --- | --- |
|  | CO_2_ | PDB_standard_ | | CH_4_ | VPDB_standard_ | |
| CO_2_ from the MxC_AC_ |  | -56.4 | -58.4 |  |  |  |
| CH_4_ fed to  the MxC_AC_ |  |  |  |  | -37.14 | -37.23 |

Where δ = (R_Sample_/R_Standard_ − 1) × 1000‰ and *R*= ^13^C/^12^C

**Table S3.** Summary of the metagenome data of the AOM-EET samples

| **Characteristics** | **Paired-end reads** | **Merged reads** | **QC reads** |
| --- | --- | --- | --- |
| Size (gigabases) | 4.998 | 4.966 | 3.205 |
| Sequences | 49,491,596 | 48,767,523 | 32,370,177 |
| Mean sequence length (bases) | 101 ± 0 | 101 ± 7 | 99 ± 13 |

**Table S4.** The number of abundance in AOM and EET-related genes

| **Function** | **Gene** | **Reads** |
| --- | --- | --- |
| anaerobic oxidation of methane  (AOM)^*^ | methyl-coenzyme M reductase  (Mcr) | 4,266 |
|  | Tetrahydromethanopterin S-methyltransferase  (Mtr) | 2,663 |
|  | methylenetetrahydromethanopterin reductase  (Mer) | 737 |
|  | F420-dependent methylenetetrahydromethanopterin dehydrogenase  (Mtd) | 576 |
|  | N(5),N(10)-methenyltetrahydromethanopterin cyclohydrolase  (Mch) | 943 |
|  | formylmethanofuran-tetrahydromethanopterin formyltransferase  (Ftr) | 1,322 |
|  | formylmethanofuran dehydrogenase  (Fmd) | 6,220 |
| Extracellular electron transfer  (EET)^**^ | c-type cytochrome | 85,601 |
|  | type IV pili | 15,188 |
|  | formate dehydrogenase | 15,273 |
|  | hydrogenase expression/formation protein | 2,361 |

^*^The number of total sequences is 5,101,250 based on KEGG Orthology (KO) by the hierarchical classification tool in MG-RAST.

^**^The number of total sequences is 9,013,251 based on KEGG by the all annotation tool in MG-RAST.
